# Diet-induced β-cell insulin resistance results in reversible loss of functional β-cell mass

**DOI:** 10.1096/fj.201800826R

**Published:** 2018-06-29

**Authors:** Meike Paschen, Tilo Moede, Ismael Valladolid-Acebes, Barbara Leibiger, Noah Moruzzi, Stefan Jacob, Concha F. García-Prieto, Kerstin Brismar, Ingo B. Leibiger, Per-Olof Berggren

**Affiliations:** The Rolf Luft Research Center for Diabetes and Endocrinology, Karolinska Institutet, Stockholm, Sweden

**Keywords:** *in vivo* imaging, diabetes mellitus, fluorescence microscopy, biosensor, diet intervention

## Abstract

Although convincing in genetic models, the relevance of β-cell insulin resistance in diet-induced type 2 diabetes (T2DM) remains unclear. Exemplified by diabetes-prone, male, C57B1/6J mice being fed different combinations of Western-style diet, we show that β-cell insulin resistance occurs early during T2DM progression and is due to a combination of lipotoxicity and increased β-cell workload. Within 8 wk of being fed a high-fat, high-sucrose diet, mice became obese, developed impaired insulin and glucose tolerances, and displayed noncompensatory insulin release, due, at least in part, to reduced expression of syntaxin-1A. Through reporter islets transplanted to the anterior chamber of the eye, we demonstrated a concomitant loss of functional β-cell mass. When mice were changed from diabetogenic diet to normal chow diet, the diabetes phenotype was reversed, suggesting a remarkable plasticity of functional β-cell mass in the early phase of T2DM development. Our data reinforce the relevance of diet composition as an environmental factor determining different routes of diabetes progression in a given genetic background. Employing the *in vivo* reporter islet–monitoring approach will allow researchers to define key times in the dynamics of reversible loss of functional β-cell mass and, thus, to investigate the underlying, molecular mechanisms involved in the progression toward T2DM manifestation.—Paschen, M., Moede, T., Valladolid-Acebes, I., Leibiger, B., Moruzzi, N., Jacob, S., García-Prieto, C. F., Brismar, K., Leibiger, I. B., Berggren, P.-O. Diet-induced β-cell insulin resistance results in reversible loss of functional β-cell mass.

Type 2 diabetes (T2DM) is a heterogeneous disease that has reached pandemic dimensions. The development of T2DM is influenced by genetic and environmental factors; hence, each patient has unique genetics and susceptibility to environmental factors. Consequently, there is a huge variety in the underlying mechanisms leading to manifestation of the disease. Therefore, there is an absolute need to understand the interface between genetics and environment in T2DM at the individual level and, based on that understanding, to establish well-defined and personalized programs for prevention and treatment of the disease.

Genome-wide association studies revealed that most identified, potential genes associated with T2DM development are linked to pancreatic islet/β-cell function (1). To that end, pancreatic islet/β-cell insulin signaling has been proposed to be of importance for appropriate β-cell function and survival. Consequently, β-cell insulin resistance is likely to constitute an important factor causing and/or contributing to β-cell dysfunction and T2DM development (2–4). However, because most of the data have been obtained from genetic models, including both β-cell–targeted manipulation of candidate genes (5–11) and models of insulin resistance and obesity (12–15), the pathophysiologic relevance of β-cell insulin resistance in diet-induced T2DM development remains unclear.

Diabetes manifests when β cells cannot compensate for the increasing demand in insulin to maintain euglycemia. Although extensive efforts have been made to monitor the dynamics of total β-cell mass during diabetes development (16), there is a lack of techniques that allow longitudinal monitoring of functional β-cell mass. We have previously shown that pancreatic islets transplanted to the anterior chamber of the eye (ACE) can be used to monitor islet cell function and survival *in vivo* noninvasively and longitudinally with cellular resolution by confocal laser scanning microscopy (17, 18). Importantly, islets engrafted in the ACE report on the function of *in situ* pancreatic islets in the same animal (13, 14, 19–24).

In the present study, we wanted to test the hypothesis that diet-induced β-cell insulin resistance intersects with genetics and specifically affects functional β-cell mass and, thereby, the development of T2DM. We reproduced Western-style, fast-food consumption by treating diabetes-prone, C57Bl/6J mice with a combination diet consisting of solid high fat and liquid sucrose (HFHSD) or fructose (HFHFrD) and monitored *in vivo* β-cell insulin sensitivity noninvasively with reporter islets in the ACE expressing a β-cell–specific, insulin-resistance biosensor (13). To evaluate the consequences for functional β-cell mass, we employed a combination of reporter islets that allowed *in vivo* monitoring of glucose-induced β-cell reporter gene transcription as well as Ca^2+^ handling during the course of the diet-intervention study.

## MATERIALS AND METHODS

### Animals and diet

Male, C57Bl/6J (B6) mice were purchased at 2 mo old from Charles River Laboratories (Wilmington, MA, USA). After delivery, the mice were allowed to adapt to the animal core facility for 1 wk before the start of the experiment. All mice were group-housed on a 12/12-h dark/light cycle with free access to food and water. If not otherwise stated, the mice received a normal chow diet (R70; Lantmännen, Stockholm, Sweden). The following special diets were fed to 3-mo-old B6 mice for 8 wk: a high-sucrose diet (HSD; 32% sucrose dissolved in tap water); a high-fat diet (HFD; 60% kcal from fat, TD.06414; Envigo, Huntingdon, United Kingdom); a HFHSD (HFD + HSD); a HFHFrD [HFD + high-fructose diet (HFrD; 32% fructose in tap water)]; and a control diet [managed formulation–purified ingredient diet (5P76; LabDiet, St. Louis, MO, USA)]; with tap water. The control diet given to the control group differed from the R70 diet all animals received before and some mice received up to 8 wk after the diet intervention. Consequently, all the animals underwent the same stress provoked by diet change. All experiments were performed in accordance with the Karolinska Institutet’s guidelines for the care and use of animals in research and were approved by the institute’s Animal Ethics Committee.

### Expression constructs

The adenovirus assembly encoding the β-cell insulin resistance biosensor (βIRB) was previously described in Paschen *et al.* (13). The adenovirus encoding the β-cell fluorescent metabolic transcriptional-response indicator (βFLUOMETRI) was generated as follows: pENTR1A.RIP1.DsRed2/rbGK.EGFP/CMV.Cerulean was generated by inserting the RIP1.dsRed2/rbGK.EGFP cassette from pd2.RIP1.dsRed2/rbGK.EGFP (25) into pENTR1A (Thermo Fisher Scientific, Waltham, MA, USA) and adding a CMV.Cerulean cassette downstream of the rbGK.EGFP cassette. The 3 individual expression cassettes are separated by transcription blocker sequences from the pd2EGFP-Promoter (Takara Bio, Kusatsu, Japan). All constructs were verified by DNA sequencing. The RIP1.DsRed2/rbGK.EGFP/CMV.Cerulean cassette was transferred into the promoterless adenovirus plasmid pAd/PL-DEST (Thermo Fisher Scientific) by the Gateway technique. The ViraPower Adenoviral Expression System (Thermo Fisher Scientific) was used to generate a replication-deficient adenovirus, which was used for transduction of cells and islets.

### Isolation and transduction of pancreatic islets

Islets were prepared from mice by duct injection of collagenase (F. Hoffmann-La Roche, Basel, Switzerland) and were handpicked under a stereomicroscope MZ6 (Leica Microsystems, Wetzlar, Germany) after digestion (18). Thereafter islets were cultured in RPMI-1640 medium, with a final concentration of 10% heat-inactivated fetal bovine serum, 2 mM glutamine, 100 U/ml penicillin, and 100 µg/ml streptomycin at 5% CO_2_ and 37°C. Islets were transduced with 10^7^ plaque-forming units/ml of the respective expression construct encoding the adenovirus.

### Transplantation of pancreatic islets into the ACE

If not otherwise stated, 2–3 d after transduction, islets were transplanted into the ACE of syngeneic, age-matched, littermate recipients, using a technique previously described by Speier *et al.* (18). Briefly, under anesthesia, islets of Langerhans were transplanted into the ACE with a glass cannula after generating a puncture in the cornea with a 27-gauge needle. Great care was taken to avoid bleeding and damage to the iris. Mice were injected s.c. with Temgesic (0.1 ml/kg; RB Pharmaceuticals, Berkshire, United Kingdom) for postoperative analgesia. The first transplantation was performed at 2 mo of age and, if necessary, was retransplanted after 2 mo.

### *In vivo* imaging of intraocular islet grafts

Islet grafts were imaged *in vivo* beginning 1 mo after transplantation (17, 18) and at 0, 4, and 8 wk after the start of diet intervention, as well as up to 8 wk after refeeding a chow diet. An upright laser scanning confocal microscope (TCSSP5; Leica Microsystems), equipped with a long-distance, water-dipping objective (HXC-APO10×/0.30 numerical aperture; Leica Microsystems) and a custom-built stereotaxic head holder, allowing positioning of the mouse eye containing the engrafted islets toward the objective used. Viscotears (Théa Nordic, Örebro, Sweden) was used as an immersion liquid between the eye and the objective, and isoflurane was used to anesthetize the mice during *in vivo* imaging. Islets were imaged as 3-dimensional stacks with 3-µm step size. After imaging, the mice were allowed to recover from anesthesia, and an i.p. glucose tolerance test (IPGTT) was performed; 4 h after glucose injection, a second imaging was performed using the same settings as described above to obtain the 4-h image set.

#### Pancreatic βIRB

Enhanced green fluorescent protein (EGFP) was excited at 488 nm, and the fluorescence was detected at 505–535 nm. Tomato was excited at 561 nm, and fluorescence was detected at 580–650 nm. Backscatter signal (26) from the 561-nm excitation was collected at 555–565 nm.

#### βFLUOMETRI

Cerulean fluorescence was excited at 405 nm and detected at 460–490 nm. EGFP fluorescence was excited at a 488 nm and detected at 505–535 nm. Red fluorescent protein (DsRed2) was excited at 561 nm and detected at 580–650 nm. Backscatter signal (26) from the 561 nm excitation was collected at 555–565 nm. Channel crosstalk was avoided by using in-between lines sequential imaging, combining Cerulean, backscatter, and dsRed2 signals and separating the EGFP signal.

### Image analysis

#### βIRB

*In vitro* and *in vivo* imaging were analyzed with ImageJ (U.S. National Institutes of Health, Bethesda, MD, USA) and the Leica Application Suite software (Leica Microsystems), respectively. For each cell, the central plane was determined with information from each detection channel (EGFP, Tomato, and backscatter); 3 regions of interest were drawn manually for each cell: nucleus, cytoplasm, and background, respectively (13). Only the intensity values of the EGFP channel were considered for the calculation of the relative βIRI (rβIRI): for each cell, a ratio was calculated based on the obtained fluorescence intensity values: ratio *r* = (Nucleus − Background)/(Cytoplasm − Background). For each experimental *in vitro* and *in vivo* condition, the ratio was calculated. For *in vivo* experiments, the mean ratio of the experimental starting point (*t* = 0; correction value *c*) was used to further calculate the rβIRI for each time point and experimental group: rβIRI = *r*/*c*. More precisely, in the diet-intervention study, the mean ratio of all mice at 0 wk of treatment was the correction value. The rβIRI was considered a measure of β-cell insulin resistance and was used for further statistical analysis. For *in vitro* experiments, the mean ratio of the control group for the respective time point (correction value *c*) was used to further calculate the rβIRI for each time point and experimental group: rβIRI = *r*/*c*.

#### βFLUOMETRI

Image analysis for *in vitro* experiments was performed as described in (27–30) using ImageJ. Average fluorescence intensities for each cell was determined for *t* = 60 min (start) and *t* = 240 min. *In vivo* imaging results were analyzed with Leica Application Suite software. For each analyzed cell, fluorescence intensity for all 3 fluorescent dyes (Cerulean, EGFP, and DsRed2) was determined before glucose stimulation and 4 h after glucose stimulation. To calculate the change in promoter activity after glucose stimulation, as a readout for β-cell functionality, fluorescence intensity for each cell was determined at the beginning of the experiment and 4 h after glucose stimulation. Cells were identified with the DsRed2-fluorescence signal, thereby ensuring the analysis of β cells. Promoter activation was calculated as follows: [(EGFP/DsRed2_4-h_ − Background_4-h_)/(Cerulean_4-h_ − Background_4-h_)]/[(EGFP/DsRed2_start_ − Background_start_)/(Cerulean_start_ − Background_start_)]. To determine functional β-cell mass as a percentage of glucose-responsive cells, cells with promoter activation >1.15 were considered responsive.

### Backscatter intensity

Backscatter intensity was analyzed in the backscatter images obtained during the imaging of the βFLUOMETRI biosensor with Image J. Average signal intensity of the islet was normalized to the average signal intensity of the surrounding iris and the ratio was used as backscatter intensity.

### Islet size

Islet size was determined based on the backscatter images obtained during the imaging of the βFLUOMETRI biosensor, as described in Van Krieken *et al.* (31). Relative islet size was calculated by dividing the size at 8 wk by the size at the start of the experiment for each individual islet.

### Calcium imaging

For Ca^2+^ measurements, islets from C57Bl/6J mice, heterozygous for Ins-CRE and floxed genetically encoded calcium indicator 3 (GCaMP3) (32, 33), were isolated and used for transplantation. From 4 wk after transplantation, the imaging was performed as follows: unfed mice were anesthetized with midazolam/fentanyl/fluanisone (i.p.), and during imaging, they were supplied with oxygen *via* a nose mask. The whole microscope was enclosed to allow heating to 35°C, with the microscope equipped with a ×25/0.95 numerical aperture objective. GCaMP3 was excited at 480 nm, and fluorescence detected at 500–550 nm. A tail vein catheter containing 14 µl heparin (100 IU/ml) was inserted in the mouse’s tail, and heparin was injected; 2 min after the start of imaging, glucose (0.4 g/kg) was carefully injected. Images were acquired with μManager microscopy software (34). Image processing was performed in MatLab (MathWorks, Natick, MA, USA). Every *z*-stack was denoised (35), and consecutive planes were aligned (36). *z*-Stacks were deconvolved (37) and registered to a reference *z*-stack (38). Individual β cells were identified based on their temporal [Ca^2+^]_i_ profile. Single-cell [Ca^2+^]_i_ traces were normalized to their baseline. β cells were considered as having responded when their glucose-induced [Ca^2+^]_i_ increase was >3 times the sd before glucose injection.

### IPGTT

To determine glucose tolerance, blood glucose levels were measured in mice that were unfed for 6 h at basal state (0 min) and at 5 (not always measured), 10, 30, 60, and 120 min after glucose injection (2 g/kg body weight i.p., dissolved in PBS). The results were depicted as the area under the curve (AUC) of the IPGTT. Glucose concentrations were measured with the Accu-Chek Aviva monitoring system (F. Hoffmann-La Roche).

### Intraperitoneal insulin tolerance test

To measure whole-body insulin resistance, an intraperitoneal insulin tolerance test (IPITT) was performed to determine the rate of glucose blood levels after an insulin challenge. Blood glucose concentration was measured in mice that were unfed for 6 h at basal state (0 min). Then, mice were injected with insulin (0.25 U/kg body weight i.p., diluted in PBS; Novo Nordisk, Bagsværd, Denmark), followed by glucose administration (1 g/kg body weight i.p.), and blood-glucose concentrations were determined at 15, 30, 60, 90, and 120 min after glucose injection. The results are depicted as the AUC of the IPITT.

### Intraperitoneal pyruvate tolerance test

To determine liver insulin tolerance, blood glucose levels were measured in mice unfed for 12 h overnight at basal state (0 min) and at 30, 60, 90, 120, and 180 min after pyruvate injection (2 g/kg body weight i.p., dissolved in PBS). The results are depicted as the AUC of the intraperitoneal pyruvate tolerance test (IPPTT).

### Body weight and fasting blood glucose

Body weight and fasting blood glucose were measured after 6 h denial of food.

### Serum biology

Blood samples were obtained at all time points during the IPGTT, centrifuged to gain blood serum, and preserved at −20°C until use. Ultrasensitive mouse ELISA kits (Crystal Chem, Elk Grove Village, IL, USA) were used to analyze different factors in the serum.

### β-cell proliferation measurement with 5-bromo-2′-deoxyuridine

To assess β-cell proliferation *in vivo*, mice were treated with 0.8 mg/ml of the synthetic nucleoside and thymidine analog 5-bromo-2′-deoxyuridine (BrdU; Thermo Fisher Scientific) 1 wk during the second month of diet treatment. After isolation and sectioning, pancreases were stained for BrdU and insulin to determine the amount of proliferating β cells. The images obtained with the Pathway 855 imaging system (BD Biosciences, San Jose, CA, USA) were analyzed with the Cell Profiler (http://cellprofiler.org/). Nuclei were identified by DAPI staining; the intensities of the insulin and BrdU staining were measured in the nucleus and displayed as histogram. Insulin-positive objects were defined as β cells. BrdU-positive and -negative cells were identified and used for calculating the percentage of BrdU-positive β cells.

### Tissue extraction and sectioning

Pancreas tissue was obtained and sectioned to verify and complement data obtained *in vivo*. Mice were anesthetized with isoflurane and transcardially perfused with PBS, followed by freshly prepared 4% (wt/vol) paraformaldehyde in PBS. Pancreases were dissected and postfixed for 2 h. Before cryopreservation, the tissues were processed with a sucrose gradient [10–30% (wt/vol) sucrose in PBS containing 0.01% (wt/vol) sodium azide and 0.02% (wt/vol) bacitracin], frozen in dry ice, and preserved at −80°C until use. Then, 20-µm-thick pancreas and liver cryosections were collected on SuperFrost Plus microscope slides (VWR International, Radnor, PA, USA) and kept at −20°C until use.

### Immunofluorescence in pancreas tissue

For immunostaining, pancreas tissue sections were equilibrated to room temperature, washed, blocked, and then, incubated with the primary antibodies (anti-FoxO1 and anti-insulin; Cell Signaling Technology, Danvers, MA, USA) in the presence of 0.1% Triton X-100 and 10% serum. After washing, secondary antibodies were applied, and mounting with medium containing DAPI for nuclear counterstaining (Thermo Fisher Scientific) was performed after repeated washing.

### Western blotting

Islets were lysed immediately after isolation in lysis buffer [50 mM Tris (pH 7.5); 1 mM EDTA; 1 mM EGTA; 0.5 mM Na_3_VO_4_; 0.1% (v/v) 2-mercaptoethanol; 1% Triton X-100; 50 mM NaF; 5 nM sodium pyrophosphate; 10 mM sodium β-glycerol phosphate; 0.1 mM PMSF; 1 μg/ml of aprotinin, pepstatin, and leupeptin; and 1 μM microcystin]. The proteins (45 μg) were separated over a 7.5% SDS-polyacrylamide gel (Laemmli buffering system) and electrotransferred to a PVDF membrane. In the cases of phospho-specific antibodies, the membranes were probed with the respective phospho-specific antibodies, then stripped and reprobed with antibodies recognizing the respective total protein levels. The following antibodies were used: rabbit pAb phospho-FoxO1 (Ser256), rabbit mAb FoxO1, rabbit mAb phospho-insulin receptor β (Tyr 1150/1151), rabbit mAb insulin receptor β, rabbit mAb phospho-Akt (Ser 473), rabbit pAb pan-Akt, and rabbit mAb Syntaxin-1A, which were all purchased from Cell Signaling Technology; and mouse mAb α-tubulin (MilliporeSigma, Billerica, MA, USA). Immunoreactivity was detected with horseradish peroxidase–conjugated secondary antibodies using Clarity Western ECL substrate and the Chemi Doc Touch imaging system (Bio-Rad Laboratories, Hercules, CA, USA). Band intensities were quantified with Image Lab (v.5.2.1) software from Bio-Rad Laboratories.

### Gene expression analysis in islets of Langerhans

For expression analysis, 100–200 freshly isolated islets were processed with RNeasy Mini Kit (Qiagen, Hilden, Germany) to isolate mRNA. cDNA was synthesized with an RT^2^ First Strand Kit (Qiagen) and used in a SYBR Green–based custom PCR array. Analysis was performed as previously described by Schmittgen *et al.* (39), where the cycle threshold (2^−Δ^*^Ct^*) was calculated for experimental groups and used for further statistical analysis.

### Statistics

The values are expressed as means ± sem. A 2-sided, unpaired *t* test was used to determine statistical significance among different treatment groups. Statistical significance was used as follows: *P < 0.05, **P < 0.01, ***P < 0.001. Origin 2015 64-bit (OriginLab, Northampton, MA, USA) and Excel (Microsoft, Redmond, WA, USA) were used for statistical analyses. The 2-sided, unpaired *t* test was used to determine statistical significance between treatment groups and the control group.

## RESULTS

### The combination of HFD and HSD, but not HFD and HFrD, leads to β-cell insulin resistance

We transplanted reporter islets expressing the β-cell insulin resistance biosensor βIRB (13) to the ACE into 2-mo old male C57Bl/6J (B6) mice, let the islets engraft for 1 mo, and obtained fluorescence from the biosensor by confocal microscopy. We determined β-cell insulin sensitivity by calculating the relative β-cell insulin resistance index (rβIRI; see Materials and Methods). We administrated a solid HFD in combination with either an HSD (HFHSD) or an HFrD (HFHFrD). Throughout the study, we compared the diet intervention groups with mice fed a control diet and tap water, monitoring the dynamics of insulin sensitivity and additional physiologic parameters. Whole-body insulin resistance, representing primarily insulin resistance in muscle and fat, was assessed by an IPITT. To measure liver insulin sensitivity *in vivo* an IPPTT was used, which is a measurement of insulin-sensitive gluconeogenesis of the liver (40). *In vivo* glucose handling was assessed by an IPGTT.

**Figure 1:**
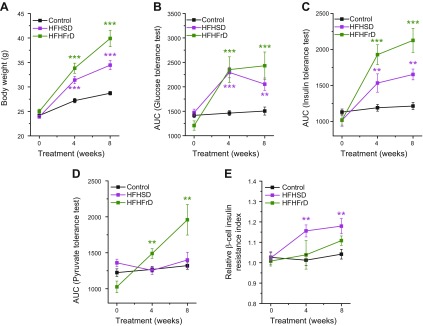
The combination of HFD and HSD, but not HFD and HFrD, leads to β-cell insulin resistance. *A*) Body weight of mice fed a control diet, HFHSD, or HFHFrD (*n* = 5–11). *B*) Glucose tolerance obtained by IPGTT and depicted as AUC of the IPGTT in mice fed a control diet, HFHSD, or HFHFrD (*n* = 5–10). *C*) Whole-body insulin tolerance obtained by IPITT and depicted as AUC of the IPITT in mice fed a control diet, HFHSD, or HFHFrD (*n* = 5–9). *D*) Liver insulin tolerance obtained by IPPTT and depicted as AUC of the IPPTT in mice fed a control diet, HFHSD, or HFHFrD (*n* = 5–8). *E*) Relative β-cell insulin resistance measured using the β-cell insulin resistance biosensor (βIRB) in islets transplanted into the ACE in mice fed a control diet, HFHSD, or HFHFrD (*n* = 4–14). *P < 0.05,**P < 0.01, ***P < 0.001. Data are expressed as means ± sem. See also Supplemental Fig. S1.

Mice fed an HFHSD had an increased body weight (**Fig. 1*A***) and showed an impaired glucose tolerance (Fig. 1*B* and Supplemental Fig. S1) 4 and 8 wk after start of diet intervention. This was paralleled by whole-body insulin resistance (Fig. 1*C* and Supplemental Fig. S1) and β-cell insulin resistance (Fig. 1*E*). Impaired liver insulin sensitivity (Fig. 1*D* and Supplemental Fig. S1) did not develop during the duration of the diet intervention study of 8 wk. Mice that were administered an HFHFrD showed an increased body weight (Fig. 1*A*) and impaired glucose tolerance (Fig. 1*B* and Supplemental Fig. S1). Whole-body insulin resistance was observed 4 and 8 wk after the start of the diet (Fig. 1*C* and Supplemental Fig. S1). In contrast to HFHSD, HFHFrD did not lead to β-cell insulin resistance (Fig. 1*E*) but led to impaired liver insulin sensitivity (Fig. 1*D* and Supplemental Fig. S1).

**Figure 2:**
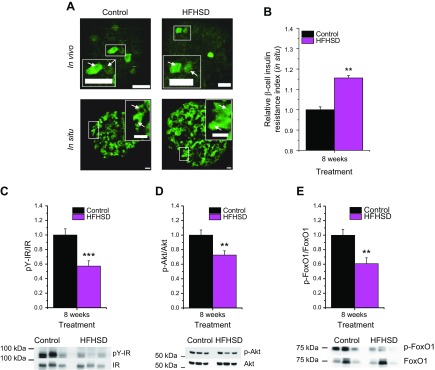
Verification of β-cell insulin resistance in HFHSD-fed mice. *A*) *In vivo* representative image of single-focal planes of engrafted islets transduced with βIRB in mice fed a control diet or HFHSD at 8 wk of diet treatment. *In situ* representative image of immunostaining of endogenous FoxO1 *in situ* in islets from pancreas sections from mice fed a control diet or HFHSD for 8 wk. Scale bars, 30 µm. *B*) Relative β-cell insulin resistance indicated by immunostaining of endogenous FoxO1 *in situ* in islets from pancreas sections from mice fed a control diet or HFHSD for 8 wk. *C*–*E*) Western blot quantification of pY-IR/IR, p-Akt/Akt, and p-FoxO1/FoxO1 in isolated islets from mice fed a control diet or HFHSD for 8 wk (*n* = 11–12). Representative Western blots for 3 control diet and HFHSD mice are shown. **P < 0.01, ***P < 0.001. Data are expressed as means ± sem. See also Supplemental Fig. S2.

To verify that the reporter islets for β-cell insulin resistance in the ACE did, indeed, report the status of the endogenous islets of the animals, we analyzed FoxO1 localization by immunofluorescence in pancreatic islet sections from mice fed an HFHSD for 8 wk (**Fig. 2*A*, *B***). The rβIRI was determined in the same way as for the images obtained from *in vivo* monitoring of reporter islets in the ACE, but, in this case, were normalized to the control animals at 8 wk. Our data show that HFHSD treatment led to β-cell insulin resistance in the endogenous islets of these animals (Fig. 2*B*). Moreover, Western blot analysis of islets from mice treated for 8 wk with HFHSD showed decreased tyrosine phosphorylation of the insulin receptor at Tyr 1150/1151 (Fig. 2*C* and Supplemental Fig. S2A, B), reduced phosphorylation of protein kinase B (Akt) at Ser 473 (Fig. 2*D* and Supplemental Fig. S2C, D), and reduced phosphorylation of FoxO1 at Ser 256 (Fig. 2*E* and Supplemental Fig. S2E, F), all indicative of impaired insulin sensitivity.

### The combination of lipotoxicity and sucrose-induced high β-cell workload leads to β-cell insulin resistance

**Figure 3:**
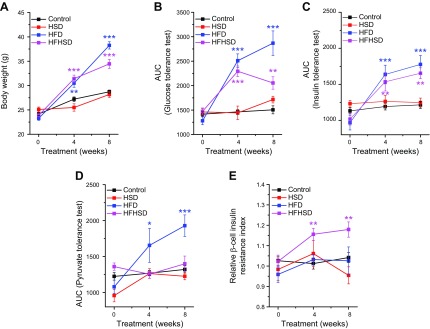
The combination of lipotoxicity and high β-cell workload leads to β-cell insulin resistance. *A*) Body weight of mice fed a control diet, HFHSD, HSD, or HFD (*n* = 4–12). *B*) Glucose tolerance obtained by IPGTT and depicted as AUC of the IPGTT in mice fed a control diet, HFHSD, HSD, or HFD (*n* = 4–12). *C*) Whole-body insulin tolerance obtained by IPITT and depicted as AUC of the IPITT in mice fed a control diet, HFHSD, HSD, or HFD (*n* = 4–9). *D*) Liver insulin tolerance obtained by IPPTT and depicted as AUC of the IPPTT in mice fed a control diet, HFHSD, HSD, or HFD (*n* = 5–8). *E*) Relative β-cell insulin resistance measured by βIRB in islets transplanted into the ACE in mice fed a control diet, HFHSD, HSD, or HFD (*n* = 3–14). *P < 0.05, **P < 0.01, ***P < 0.001. Data are expressed as means ± sem. See also Supplemental Fig. S1.

Because the HFHSD provoked β-cell insulin resistance, we examined whether the combination of fat and sucrose was necessary or whether one of those components alone was able to reproduce the same effect. To address that question, we administrated either a solid HFD or a liquid HSD to mice for 8 wk. Mice that received an HSD did not change in any of the measured physiologic parameters (**Fig. 3*A–E*** and Supplemental Fig. S1). Mice that received an HFD showed an increase in body weight (Fig. 3*A*), impaired glucose tolerance (Fig. 3*B* and Supplemental Fig. S1), whole-body insulin resistance (Fig. 3*C* and Supplemental Fig. S1), and impaired pyruvate tolerance (Fig. 3*D* and Supplemental Fig. S1). However, those mice did not develop β-cell insulin resistance (Fig. 3*E*) at 4 and 8 wk of diet intervention. These data suggest that the combination of lipotoxicity provided by the high-fat component and the high workload enforced by the high-glucose component of the diet were needed to develop β-cell insulin resistance. Consequently, we focused our further work on the HFHSD.

### Generation of a βFLUOMETRI for *in vivo* monitoring of glucose-stimulated β-cell function

**Figure 4:**
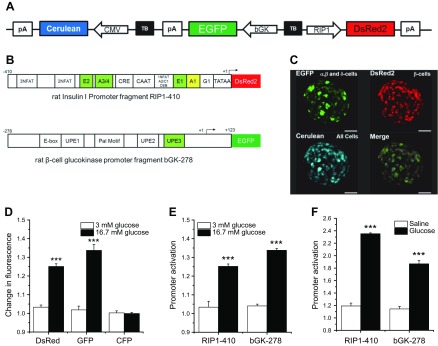
Measurement of functional β-cell mass. *A*) Schematic illustration of βFLUOMETRI. Although all cells express Cerulean under the CMV promoter, all endocrine cells express EGFP under the rat β-cell-active glucokinase promoter fragment bGK-278, and only β cells express DsRed2 under the insulin promoter fragment RIP1-410. Cerulean under the CMV promoter was used for normalization of fluorescence changes in EGFP under bGK-278 and DsRed2 under RIP1-410. *B*) Schematic illustration of the metabolic response elements within RIP1-410 and bGK-278. Elements highlighted in green are glucose and insulin responsive; that in yellow is glucose responsive, and NFAT and CRE are Ca^2+^ responsive. *C*) Representative image of an islet expressing βFLUOMETRI (*n* = 6). Scale bars, 50 µm. *D*) Changes in EGFP, DsRed2, and Cerulean (CFP) fluorescence after glucose stimulation in islets *in vitro* (*n* = 6). *E*) Promoter activation after glucose stimulation *in vitro*. DsRed2 and EGFP fluorescence intensities were normalized to CFP fluorescence intensity (*n* = 6). *F*) Activation of RIP1-410 and bGK-278 after glucose and saline injection *in vivo* (*n* = 5). ***P < 0.001. Data are expressed as means ± sem. See also Supplemental Fig. S3.

To monitor the consequences of HFHSD longitudinally, and thus the β-cell insulin resistance, on β-cell function *in vivo*, we again used the ACE *in vivo* imaging approach. We developed a biosensor that allowed us to monitor β-cell function in response to glucose stimulation by changes in fluorescence intensity. As we have previously shown (27–29, 41, 42), the rat insulin gene-1 promoter (RIP1) and the rat β-cell–active glucokinase gene promoter contain metabolic response elements that allow an increase in promoter activity upon stimulation with glucose or insulin *in vitro*. To generate βFLUOMETRI for *in vivo* monitoring, we combined 3 expression cassettes in a single adenoviral vector (**Fig. 4*A–C***). The first cassette comprises a DsRed2 gene driven by a fragment of the rat insulin-1 gene promoter (−410/+1 bp; RIP1-410), containing transcriptional metabolic response elements for glucose, insulin, Ca^2+^, and cAMP as well as *cis*-elements allowing β-cell–selective expression (27, 43–45). The second cassette comprises the EGFP gene driven by a fragment of the rat β-cell–active glucokinase gene promoter (−278/+123 bp; bGK-278) containing transcriptional metabolic response elements for glucose and insulin, the latter being activated by a signaling cascade that is different from the one activating the RIP1-410 fragment (Supplemental Fig. S3A) (28, 46). As a third expression cassette, we chose cytomegalovirus (CMV)-promoter–driven Cerulean, which does not respond to the metabolic stimuli glucose or insulin (27).

To verify the functionality of βFLUOMETRI, we transduced mouse islets with an adenovirus encoding the biosensor and first analyzed changes of fluorescence intensities of DsRed2, EGFP, and Cerulean upon glucose stimulation *in vitro*. Fluorescence intensities were analyzed only in DsRed2-positive cells, that is, β cells. Glucose stimulation led to a significant increase in both EGFP and DsRed2 fluorescence, whereas fluorescence of CMV-promoter–driven Cerulean remained unchanged (Fig. 4*D*). Hence, fluorescence intensity generated by CMV-promoter–driven Cerulean could be used to normalize changes in RIP1-410 and bGK-278 activities (Fig. 4*E*). To test βFLUOMETRI *in vivo*, we transduced isolated islets from B6 mice with an adenovirus encoding the biosensor and transplanted those islets to the ACE of littermates. We let the islets engraft for 1 mo before we performed the experiment, as illustrated in Supplemental Fig. S3B. Briefly, we measured fluorescence intensities of EGFP, DsRed2, and Cerulean by confocal microscopy in anesthetized mice that were unfed overnight. After the first recording, we i.p. injected awake mice with glucose or saline and performed a second recording 4 h after the injection in anesthetized mice, maintaining the previous microscopic settings. Mice injected with glucose showed a significant increase in bGK-278 and RIP1-410 activity, whereas mice injected with saline did not (Fig. 4*F*). These data showed the feasibility of using βFLUOMETRI to monitor β-cell function upon glucose stimulation *in vivo*.

### βFLUOMETRI reveals loss of functional β-cell mass in response to HFHSD and recovery after reintroduction of normal chow diet

**Figure 5:**
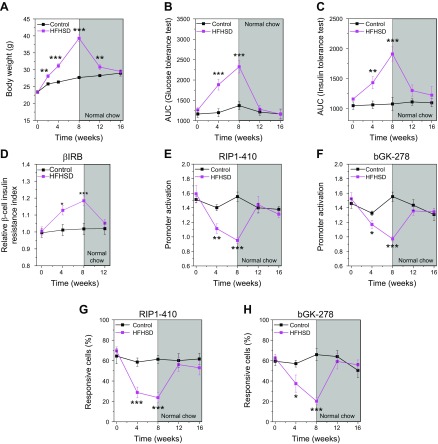
βFLUOMETRI reveals loss of functional β-cell mass in response to HFHSD and the regaining of function upon recovery from the diet. *A*) Body weight of B6 mice before, during, and after feeding of a control diet or HFHSD (*n* = 5–10). *B*) Glucose tolerance obtained by IPGTT and depicted as the AUC of the IPGTT in B6 mice before, during, and after feeding of a control diet or HFHSD (*n* = 5–10). *C*) Whole-body insulin resistance obtained by IPITT and depicted as the AUC of the IPITT in B6 mice before, during, and after feeding of a control diet or HFHSD (*n* = 5–10). *D*) Relative β-cell insulin resistance indicated by βIRB in islets transplanted into the ACE in mice before, during, and after feeding of a control diet or HFHSD (*n* = 12–20). *E*) RIP1-410 promoter activation indicated by the βFLUOMETRI and obtained *in vivo* in transduced islets transplanted into the ACE of B6 mice before, during, and after feeding of a control diet or HFHSD (*n* = 5–10). *F*) bGK-278 promoter activation indicated by the βFLUOMETRI and obtained *in vivo* in islets transplanted into the ACE of B6 mice before, during, and after feeding of a control diet or HFHSD (*n* = 5–10). *G*) Proportion of glucose-responsive cells indicated by RIP1-410 of the βFLUOMETRI and obtained *in vivo* in islets transplanted into the ACE of B6 mice before, during, and after feeding of a control diet or HFHSD (*n* = 5–10). *H*) Proportion of glucose-responsive cells indicated by bGK-278 of the βFLUOMETRI and obtained *in vivo* in islets transplanted into the ACE of B6 mice before, during, and after feeding of a control diet or HFHSD (*n* = 5–10). *P < 0.05, **P < 0.01, ***P < 0.001. Data are expressed as means ± sem.

To investigate the effect of HFHSD on β-cell function *in vivo*, we transduced isolated islets from B6 mice with an adenovirus encoding βFLUOMETRI, transplanted them to the ACE of male littermates at 2 mo old, and followed the experimental settings as above (Supplemental Fig. S3B). Within 4 wk of HFHSD the mice increased their body weight (**Fig. 5*A***) and developed impaired glucose tolerance (Fig. 5*B*), whole-body insulin resistance (Fig. 5*C*), and β-cell insulin resistance (Fig. 5*D*). All parameters became more pronounced by 8 wk of diet. Four weeks of feeding an HFHSD led to a significant reduction in glucose-stimulated activities of RIP1-410 and bGK-278 in β cells, which became more pronounced 8 wk after the start of the diet intervention (Fig. 5*E*, *F*). Because we intended to measure functional β-cell mass, we took advantage of the high amount of analyzable β cells/islets to calculate the percentage of glucose-responsive β cells (Fig. 5*G*, *H*). The data show that, under normal conditions, 64 ± 2% of the β cells were responsive to glucose stimulation. During the diet intervention with an HFHSD, the amount of glucose-responsive β cells decreased to 22 ± 3%.

To test whether β cells can recover their functionality after 8 wk of HFHSD feeding, we changed the diet to normal chow (Fig. 5). Interestingly, all measured parameters, except the body weight, recovered to normal within 4 wk of feeding normal chow diet, that is, they were not different from the control group.

### HFHSD leads to noncompensatory, glucose-stimulated insulin secretion because of a defect downstream of glucose-stimulated Ca^2+^ influx

**Figure 6:**
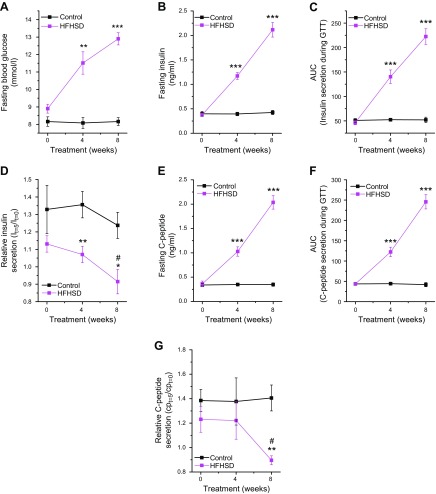
HFHSD leads to noncompensatory insulin secretion. *A*) Fasting blood glucose in mice fed a control diet or HFHSD. *B*) Fasting insulin in mice fed a control diet or HFHSD. *C*) Insulin secretion during an IPGTT in mice fed a control diet or HFHSD. *D*) Relative insulin secretion during the first 5 min of an IPGTT in mice fed a control diet or HFHSD. *E*) Fasting C-peptide results in mice fed a control diet or HFHSD. *F*) C-peptide secretion during an IPGTT in mice fed a control diet or HFHSD. *G*) Relative C-peptide secretion during the first 5 min of an IPGTT in mice fed a control diet or HFHSD. *n* = 5–6. *P < 0.05, **P < 0.01, ***P < 0.001 for control diet *vs.* HFHSD; ^#^*P* < 0.01 for HFHSD at 8 wk of treatment *vs.* 0 wk of treatment. Data are expressed as means ± sem. See also Supplemental Fig. S4.

To analyze the consequences of the HFHSD, we next analyzed the metabolic state of the animals. Mice treated with an HFHSD became overweight, had impaired glucose tolerance, were whole-body and β-cell insulin resistant (Figs. 1 and 3 and Supplemental Fig. S1) after 4 wk of diet intervention. That was paralleled by increased fasting blood glucose levels (**Fig. 6*A***), increased fasting insulin levels (Fig. 6*B*), and increased fasting C-peptide levels (Fig. 6*E*). Impairment of all parameters was more pronounced after 8 wk of HFHSD. Although the total amounts of secreted insulin (Fig. 6*C* and Supplemental Fig. S4) and C-peptide (Fig. 6*F* and Supplemental Fig. S4) were increased, the levels of insulin were not sufficient to compensate for hyperglycemia. The relative secretory response of β cells, in terms of insulin and C-peptide release during the first 5 min of an IPGTT, was significantly reduced 8 wk after the start of the diet intervention (Fig. 6*D*, *G* and Supplemental Fig. S4).

**Figure 7:**
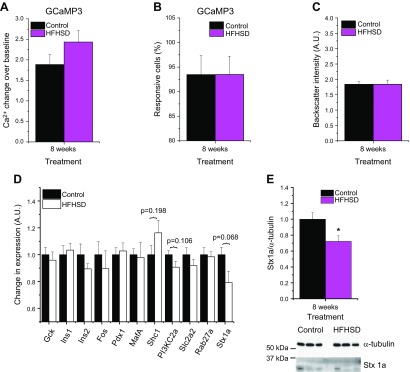
Impaired insulin secretion of HFHSD mice is due to a defect downstream of glucose-stimulated Ca^2+^ influx. *A*) Ca^2+^ excursions upon glucose stimulus, as indicated by the GCaMP3 Ca^2+^ biosensor, obtained *in vivo* in islets from Ins-Cre:GCaMP3-mice transplanted into the ACE of B6 mice at 8 wk of an HFHSD (*n* = 6–9). *B*) Amount of glucose-responsive cells indicated by the GCaMP3 Ca^2+^ biosensor obtained *in vivo* in islets from Ins-Cre:GCaMP3-mice transplanted to the ACE of B6 mice at 8 wk of an HFHSD (*n* = 6–9). *C*) Backscatter intensity of islets transplanted to the ACE of B6 mice at 8 wk of an HFHSD shown in arbitrary units (A.U.) (*n* = 10). *D*) Gene expression analysis of isolated islets from mice fed a control diet or HFHSD for 8 wk shown in A.U. (*n* = 6 mice). *E*) Western blot quantification of syntaxin-1A in isolated islets from mice fed a control diet or HFHSD for 8 wk (*n* = 11–12). Representative Western blot of 3 control diet and HFHSD mice is shown. *P < 0.05, ***P < 0.001. Data are expressed as means ± sem. See also Supplemental Figs. S5 and S6.

To better understand where in the glucose-stimulation/insulin-secretion coupling the β cells failed to secrete sufficient amounts of insulin to compensate for a glucose challenge, we next measured the glucose-stimulated β-cell Ca^2+^ handling by means of glucose-stimulated Ca^2+^ excursions in reporter islets in the ACE *in vivo*. We isolated islets from Ins-Cre:GCaMP3 mice, which express the Ca^2+^ biosensor GCaMP3 in β cells, and transplanted those islets into the ACE of male B6 mice. Eight weeks after feeding an HFHSD or control diet, Ca^2+^ excursions upon glucose stimulation were monitored in anesthetized animals by confocal microscopy. Ca^2+^ excursions were not different between the groups (**Fig. 7*A***). Similarly, the amount of responding β cells in the HFHSD group did not differ from the control group (Fig. 7*B*), suggesting a defect downstream of glucose-stimulated Ca^2+^ handling. Analysis of the backscatter intensity of the engrafted islets, indicative for insulin content (26), revealed no differences between mice fed HFHSD and the control group (Fig. 7*C*). Employing a biased transcriptomics analysis (real-time quantitative PCR) in islets obtained from male B6 mice treated for 8 wk with either a control diet or an HFHSD, we found a trend toward decreased expression of the soluble *N*-ethylmaleimide attachment receptor protein syntaxin-1A among different candidate genes implicated in β-cell function (Fig. 7*D*). The decrease in expression of syntaxin-1A was confirmed at the protein level (Fig. 7*E* and Supplemental Fig. S5).

Finally, we tested whether the HFHSD led to an increase in β-cell proliferation to compensate for inadequate function by supplementing the liquid food in both control and HFHSD mice with BrdU during wk 8 of the diet treatment. In this case, we found that HFHSD treatment led to an increase in β-cell proliferation during the last week of diet intervention (Supplemental Fig. S6A, B), which was mirrored by an increase in the relative size of the reporter islets in the ACE (Supplemental Fig. S6C).

## DISCUSSION

### Relevance of β-cell insulin resistance in dietary models of T2DM development

Sugar and fat are discussed in the context of macronutrient (over) consumption, leading to obesity, insulin resistance, and subsequent development of T2DM (47, 48). Fast food consists of food with high-fat contents and beverages with high-sugar contents, primarily sucrose or fructose. To reproduce fast-food consumption in animal models with a diabetic-prone genetic background, we performed an 8-wk diet intervention, with a combination of a solid diet high in fat and drinking water containing either sucrose (HFHSD) or fructose (HFHFrD) (49, 50) in male C57Bl/6J mice, which develop a T2DM-like phenotype when metabolically challenged (51, 52). Although both types of diet led to the development of a diabetic phenotype, including elevated fasting blood glucose levels, increased body weight, impaired glucose tolerance, and whole-body insulin resistance, only the HFHSD-treated animals developed β-cell insulin resistance in the metabolic branch of insulin signaling. Moreover, HFHFrD led to impaired pyruvate tolerance, whereas HFHSD did not. It is remarkable that none of the diets alone, that is, HFD or HSD, were able to cause β-cell insulin resistance. Even if an HFD caused functional damage to glucose and insulin handling, lipotoxicity alone was insufficient to provoke β-cell insulin resistance within 8 wk of diet treatment. In fact, the combination of lipotoxicity with the high workload of the β cell because of additional dietary glucose was required to cause β-cell insulin resistance. These data reinforce the relevance of diet composition as an environmental factor determining different routes of diabetes progression.

Western blot analysis of pancreatic islets from mice fed an HFHSD for 8 wk revealed reduced phosphorylation of Akt and FoxO1, together with reduced Tyr phosphorylation within the activation loop of the insulin receptor (Tyr 1150/1151), reflecting decreased activity by the receptor. Future work will have to show whether that is the result of enhanced activity by the phosphotyrosine phosphatases, as recently discussed for T-cell protein-tyrosine phosphatase-45 (53), or of increased binding of members of the Grb7 family to the receptor (54).

### Consequences of HFHSD and β-cell insulin resistance on functional β-cell mass

To understand how an HFHSD, and thus β-cell insulin resistance, affects β-cell function *in vivo*, we established an approach for longitudinal, noninvasive monitoring of functional β-cell mass using the reporter-islet concept. That allowed us to combine the assessment of robust parameters reflecting the metabolic state of the mice during the diet intervention, such as IPGTT, IPITT, IPPTT, fasting blood glucose, and insulin levels, with data on β-cell function by monitoring reporter islets engrafted into the ACE. These islets reported on β-cell insulin sensitivity (βIRB) and β-cell responsiveness to glucose stimulation by employing a βFLUOMETRI and a fluorescent Ca^2+^ indicator (GCaMP3). T2DM development is generally discussed as a chronic process, which, in humans, takes several years or even decades to complete. It was astonishing to see that impairments in β-cell insulin sensitivity and glucose responsiveness were already observed 4 wk after start of diet intervention and that all parameters measured became more deranged by 8 wk of HFHSD. These data support recent observations that changes in β cells occur early in response to extreme metabolic challenges (55). The many β cells expressing either βFLUOMETRI or GCaMP3, allowed us to monitor functional β-cell mass. Interestingly, although there was no change in functional β-cell mass in terms of glucose-stimulated Ca^2+^ excursions, a 65% decrease in glucose-responsive β-cell mass was observed when using the βFLUOMETRI. Remarkably, replacing the HFHSD after 8 wk of diet intervention with a normal chow diet allowed recovery of all measured parameters to within reference range. Because β cells in the reporter islets were color-coded by the biosensor, that recovery was achieved, at least in part, by the mice regaining function in the β cells that were previously malfunctioning. This highlights the plasticity of β-cell functionality, at least during the early phase of diabetes development and may have important implications for intervention of T2DM progression.

Finally, when starting to explore the molecular mechanism(s) involved in HFHSD-evoked β-cell dysfunction, we speculated that there might be a defect in insulin exocytosis based on the inability of the β cells to increase serum C-peptide levels within the first 5 min of an IPGTT. Results of a biased-transcriptomics analysis revealed no changes in the expression of genes responsible for β-cell identity (MafA, Pdx1) or genes involved in β-cell glucose handling (glucose transporter 2, glucokinase), indicating no β-cell dedifferentiation at that stage of diet intervention. This is in agreement with earlier findings by Kitamura *et al.* (56), showing that MafA may replace Pdx1 as a master regulator of β-cell identity and function when FoxO1 is enriched in the β-cell nucleus. Loss of nuclear Pdx1 and reduced binding of MafA to *cis*-elements with the promoters of respective target genes may, in addition to impaired insulin signaling, explain the loss of glucose responsiveness indicated by βFLUOMETRI under glucotoxic/lipotoxic conditions (57). Moreover, we identified reduced expression of the soluble *N*-ethylmaleimide attachment receptor protein syntaxin-1A, a key protein in insulin exocytosis (58, 59), in islets from mice fed an HFHSD for 8 wk. This is in agreement with earlier observations showing that impaired insulin signaling in β cells results in reduced expression of syntaxin-1A (8, 60). Because syntaxin-1A acts in the final steps of insulin granule docking/fusion in glucose-stimulated insulin secretion, that is, downstream of glucose-stimulated Ca^2+^ entry, these data are not in conflict with the unchanged glucose-stimulated Ca^2+^ excursions obtained by GCaMP3-reporter islets. Syntaxin-1A has been shown to bind both insulin granules and L-type voltage-gated Ca^2+^ channels (61), thus allowing glucose-stimulated Ca^2+^ influx close to the release site of the insulin granule. Previous data have demonstrated that, under conditions of lipotoxicity, the association between insulin granules and L-type voltage-gated Ca^2+^ channels is reduced, resulting in decreased insulin exocytosis despite normal glucose-evoked Ca^2+^ excursions (62, 63).

### Limitations of study

One major objective of this study was to address the pathophysiologic relevance of β-cell insulin resistance in dietary-based models of T2DM progression. To characterize the metabolic state of the mice, we used a set of techniques that were least invasive, such as excluding additional anesthetic episodes, to match them with the noninvasive character of our *in vivo* reporter-islet monitoring approach. We are well aware that for proper metabolic characterization of HFHSD-fed animals, appropriate clamp studies, as well as analysis involving metabolic cages, are necessary (40, 64). Moreover, as clearly stated, we performed a biased transcriptomic/proteomic analysis that aimed for genes that were documented to be compromised under conditions of β-cell insulin resistance. Future analyses need to be based on a nonbiased approach, including whole-RNA sequencing analysis, microRNA profiling, epigenetic characterization, and proteomic analysis, including enzyme kinetic profiling, to disclose the molecular mechanisms involved in HFHSD-based β-cell dysfunction.

## CONCLUSIONS

In summary, our data reveal that β-cell insulin resistance develops when specific dietary conditions (HFHSD) are brought onto a specific genetic background (C57Bl/6J). This highlights the relevance of diet composition as an environmental factor determining different routes of diabetes progression in a given individual with genetic predisposition for the disease. Exemplified by the HFHSD, we show that β-cell insulin resistance occurs early during T2DM progression and is provoked by the combination of lipotoxicity provided by the high-fat component and increased β-cell workload caused by the additional glucose in the diet. Within 8 wk, mice became obese, developed impaired insulin and glucose tolerances, and displayed noncompensatory insulin release, at least in part, because of reduced expression of syntaxin-1A. Reporter islets transplanted into the ACE revealed a concomitant loss of functional β-cell mass. Of note, when mice were changed from the diabetogenic diet to a normal diet, the diabetes phenotype was reversed, suggesting a remarkable plasticity of functional β-cell mass in the early phase of T2DM development. This is likely to be explained by a still functional glucose metabolism, adequate Ca^2+^ handling, and the expression of markers of β-cell identity at that stage of the disease. These findings have major implications, offering a time window within which the progression to full-blown T2DM can be halted. Finally, employing the *in vivo* reporter-islet monitoring approach will not only allow researchers to define key time points in the dynamics of reversible loss of functional β-cell mass and, thus, to investigate the underlying molecular mechanisms involved in the progression toward T2DM manifestation but also will represent a monitoring approach for a personalized medicine treatment of the disease.

## Supplementary Material

This article includes supplemental data. Please visit *http://www.fasebj.org* to obtain this information.

## Abbreviations

ACE: anterior chamber of the eye
Akt: protein kinase B
AUC: area under the curve
B6 mice: C57Bl/6J mice
BrdU: 5-bromo-2′-deoxyuridine
CMV: cytomegalovirus
DsRed: red fluorescent protein
EGFP: enhanced green fluorescent protein
βFLUOMETRI: β-cell fluorescent metabolic transcriptional response indicator
GCaMP: genetically encoded calcium indicator
HFD: high-fat diet
HFHFrD: high-fat–high-fructose diet
HFHSD: high-fat–high-sucrose diet
HFrD: high-fructose diet
HSD: high-sucrose diet
IPGTT: i.p. glucose tolerance test
IPITT: i.p. insulin tolerance test
IPPTT: i.p. pyruvate tolerance test
βIRB: β-cell insulin-resistance biosensor
RIP1: rat insulin-1 gene promoter
rβIRI: relative β-cell insulin-resistance biosensor index
T2DM: type 2 diabetes mellitus
