# Supplementary material for: Diet-induced β-cell insulin resistance results in reversible loss of functional β-cell mass
Source: FASEB J. 2018 Jun 29;33(1):204–18. doi: 10.1096/fj.201800826R (PMC6355083; doi:10.1096/fj.201800826R)
Supplement: Supplementary file 1 [file fj.201800826R.sf1.pdf]

Figure S1, related to Figure 1 and 3

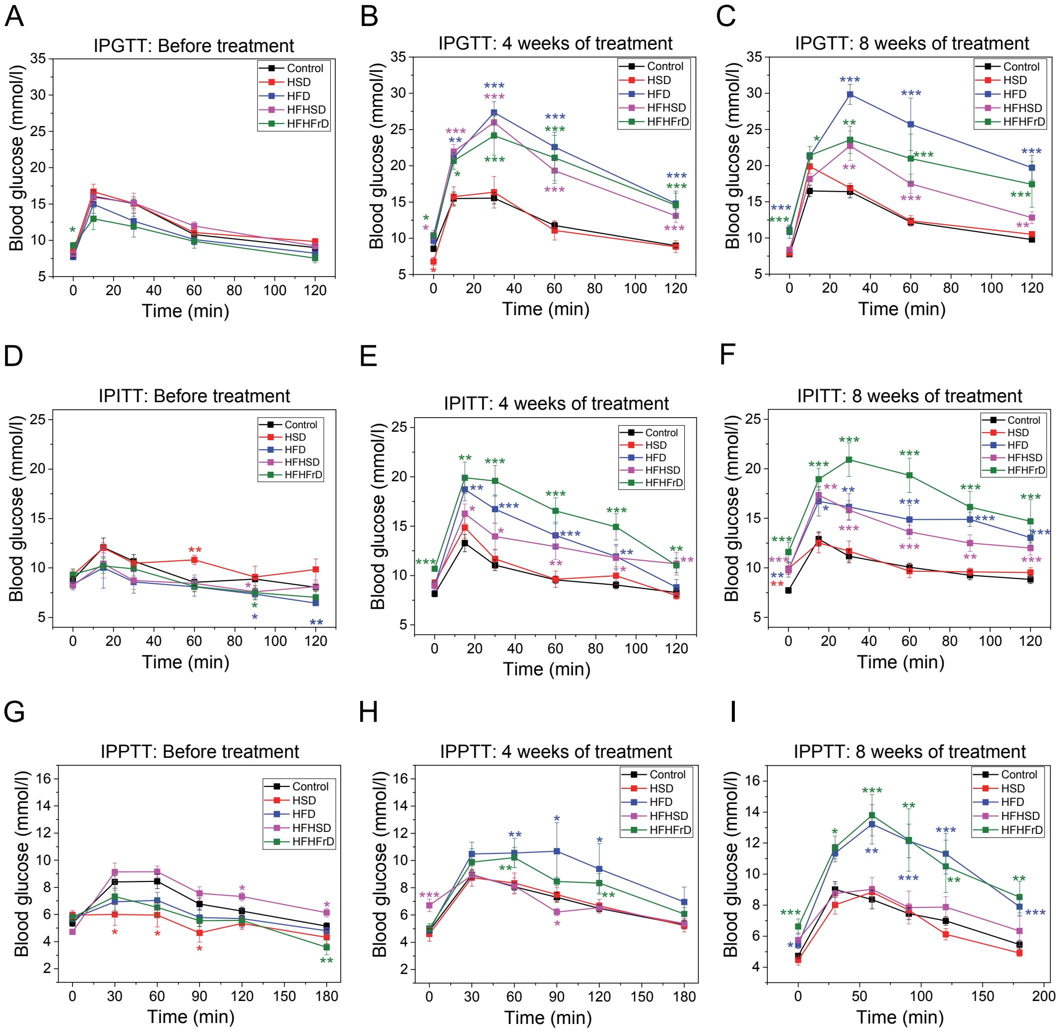

Supplemental Figure S1. Individual traces from intraperitoneal tolerance tests for each dietary condition at every treatment time point. *A-C*) Glucose tolerance at 0, 4 and 8 weeks of diet treatment obtained by intraperitoneal glucose tolerance tests (IPGTT) depicted as individual curves for each diet condition. *D-F*) Whole body insulin tolerance at 0, 4 and 8 weeks of diet treatment obtained by intraperitoneal insulin tolerance tests (IPITT) depicted as individual curves for each diet condition. *G-I*) Liver insulin tolerance at 0, 4 and 8 weeks of diet treatment obtained by intraperitoneal pyruvate tolerance tests (IPPTT) depicted as individual curves for each diet condition. $*p<0.05$; $**p<0.01$; $***p < 0.001$. Data are expressed as mean ± SEM.

Figure S2, related to Figure 2

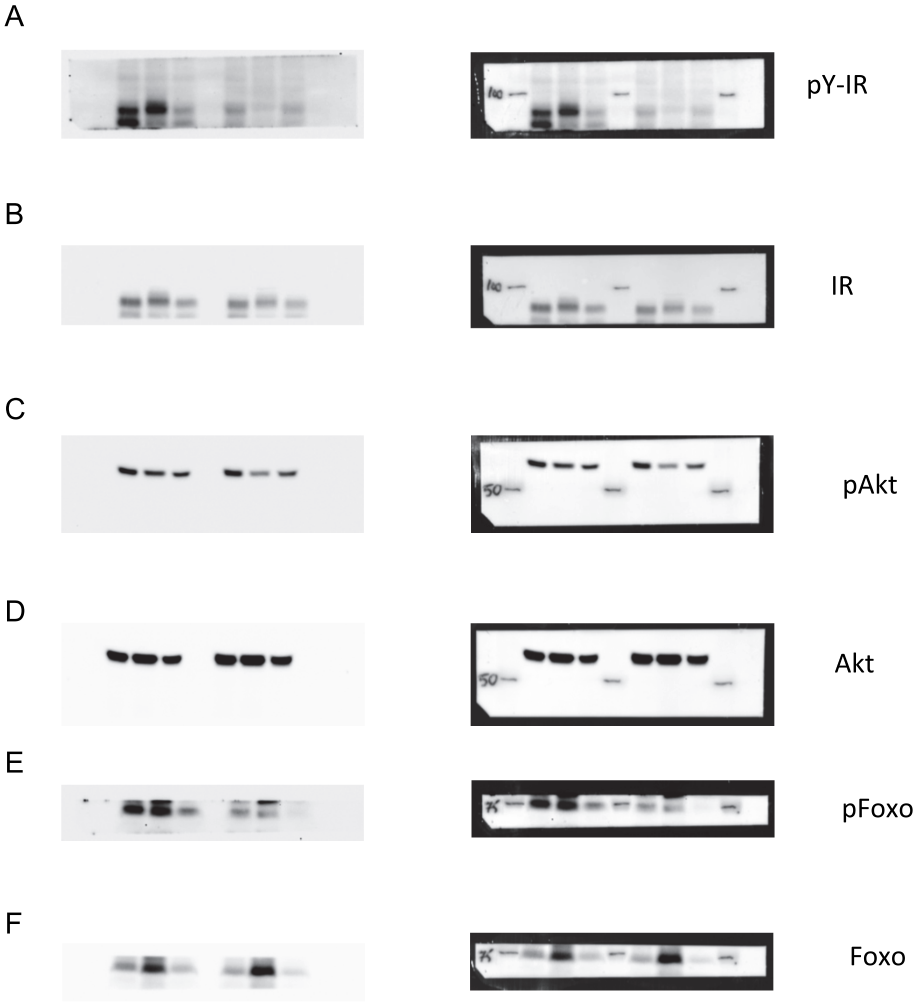

Supplemental Figure S2. Original Western blot images obtained with the Chemi Doc Touch Imaging System and the Image Lab Touch Software (BioRad). The left images were obtained with the chemiluminescence settings and the right images are merges of the chemiluminescence and colorimetric images obtained from the same piece of PVDF membrane.

Figure S3, related to Figure 4

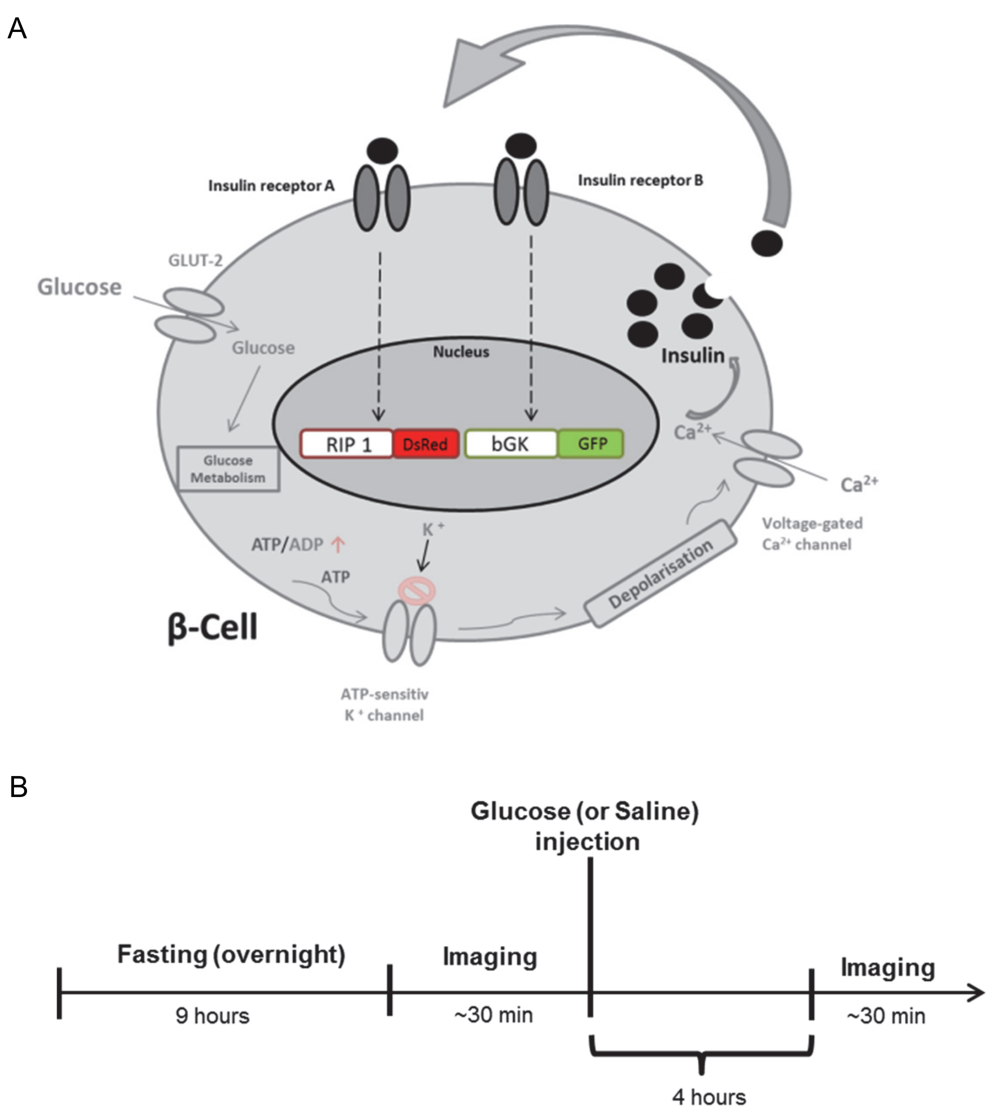

Supplemental Figure S3. Measurement of functional β-cell mass. *A*) The biosensor measures integrated β-cell function including glucose metabolism, Ca^{2+}-dependent insulin release as well as insulin signaling downstream of the two insulin receptors. *B*) Schematic illustration of the *in vivo* experiment. Mice were fasted overnight and imaged the next morning. After the imaging, awake mice were injected with glucose (2 g/kg) and imaged a second time 4 hours after the injection.

Figure S4, related to Figure 6

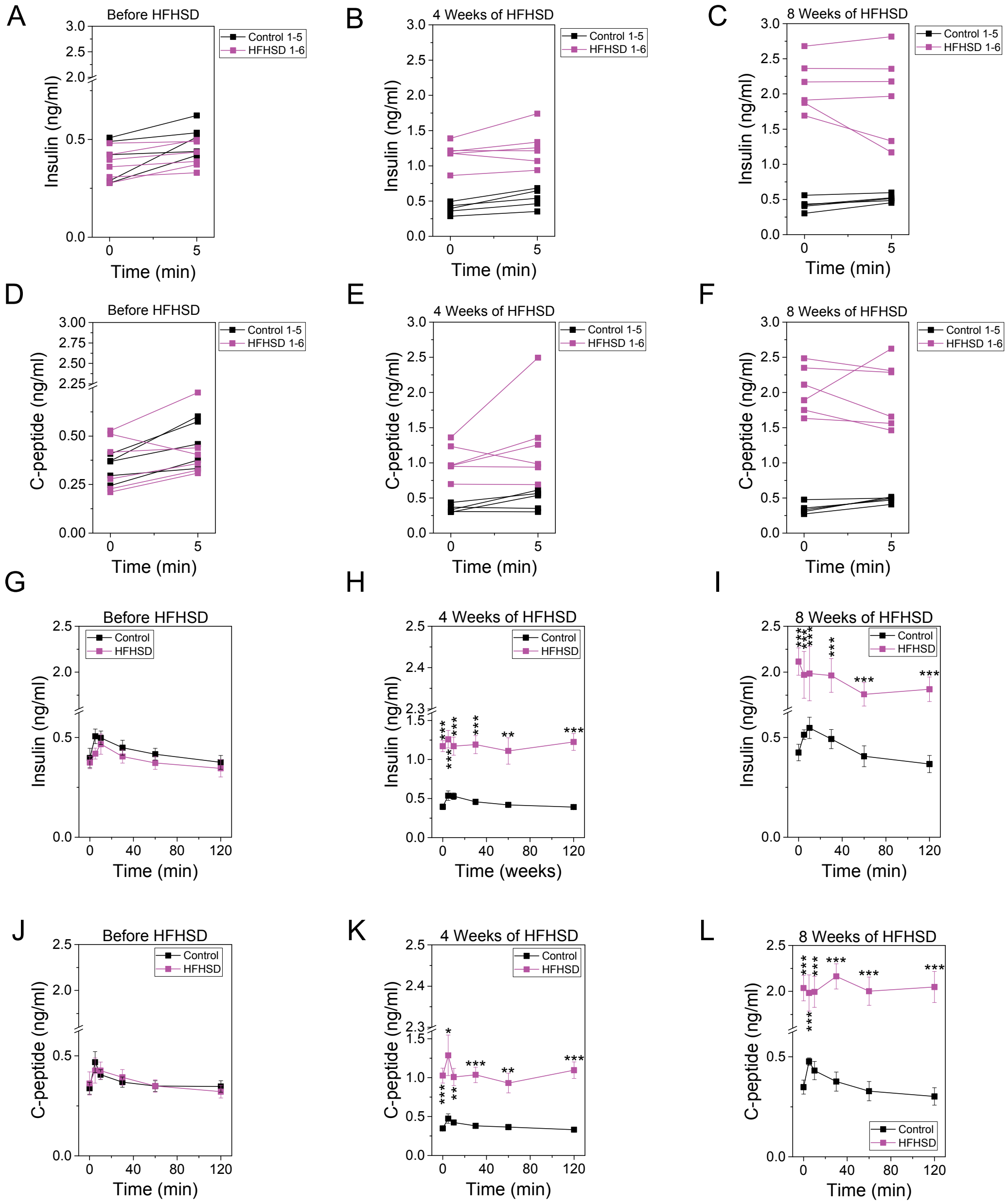

Supplemental Figure S4. Individual traces for insulin and C-peptide measurements. *A-C*) Serum insulin concentration at 0 and 5 min during the intraperitoneal glucose tolerance test (IPGTT) at 0, 4 and 8 weeks of HFHSD intervention. *D-F*) Serum C-peptide concentration at 0 and 5 min during the IPGTT at 0, 4 and 8 weeks of HFHSD intervention. *G-I*) Serum insulin concentration during the IPGTT at 0, 4 and 8 weeks of HFHSD intervention. *J-L*) Serum C-peptide concentration during the IPGTT at 0, 4 and 8 weeks of HFHSD intervention. *p<0.05; **p<0.01; ***p<0.001. Data are expressed as mean ± SEM.

Figure S5, related to Figure 7

A

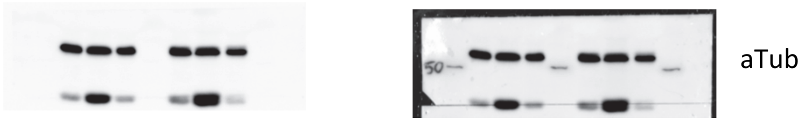

B

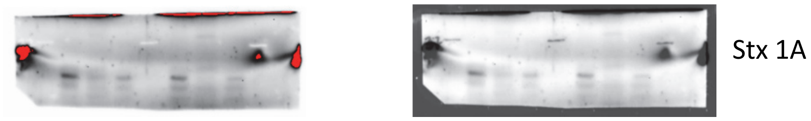

Supplemental Figure S5. Original Western blot images obtained with the Chemi Doc Touch Imaging System and the Image Lab Touch Software (BioRad). The left images were obtained with the chemiluminescence settings and the right images are merges of the chemiluminescence and colorimetric images obtained from the same piece of PVDF membrane.

Figure S6, related to Figure 7

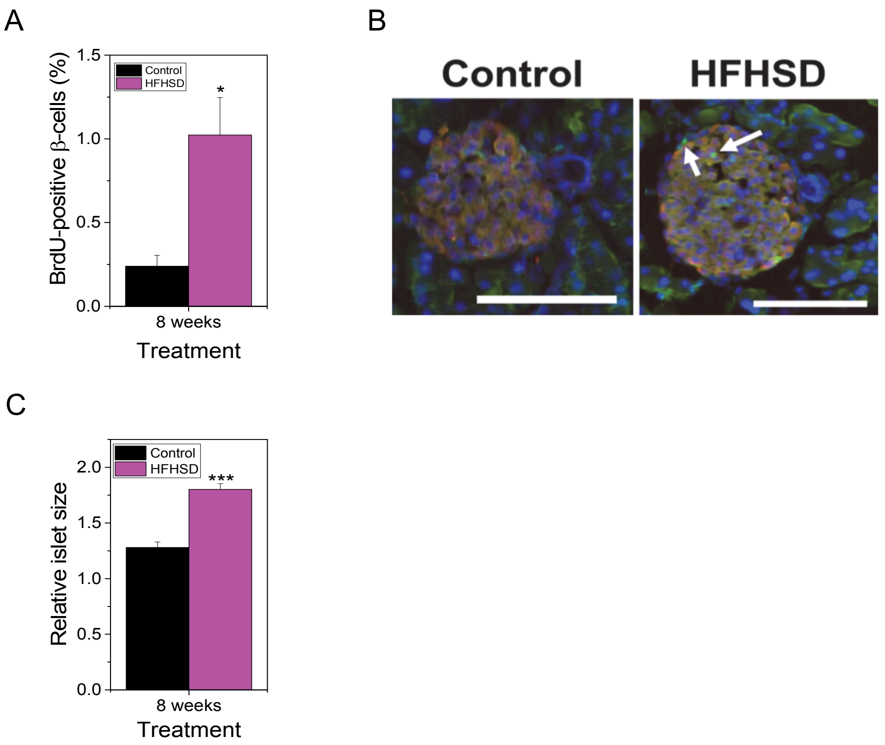

Supplemental Figure S6. Compensatory β-cell proliferation in HFHSD fed mice. (A) Percentage of BrdU-positive β-cells in pancreas sections from B6 mice fed a control diet or HFHSD for 8 weeks and receiving BrdU in the drinking water during the eights week of diet intervention (n= 3-4 mice). (B) Representative images of an islet of Langerhans from B6 mice fed a control diet or HFHSD for 8 weeks stained for BrdU (nuclear green), insulin (red) and DAPI for nuclear counterstaining (blue). Arrows indicate BrdU-positive β-cells. Scale bar: 30 µm. (C) Relative islet size of islets in the anterior chamber of the eye in B6 mice fed a control diet or HFHSD for 8 weeks (n=5-8). The two-sided, unpaired t-test was used to determine statistical significance between HFHSD and control group. *$p<0.05$; ***$p<0.001$. Data are expressed as mean ± SEM.
